# Complete mitochondrial genome of *Gampsocleis fletcheri* (Burr, 1899)

**DOI:** 10.1080/23802359.2021.1884020

**Published:** 2021-03-11

**Authors:** Lan Ma, Jinyan Chai, Zhijun Zhou

**Affiliations:** a The Key Laboratory of Zoological Systematics and Application, Hebei University, Baoding, China; b College of Life Science, Institute of Life Science and Green Development, Hebei University, Baoding, China

**Keywords:** *Gampsocleis fletcheri*, mitochondrial genome, phylogenetic analysis

## Abstract

Species of *Gampsocleis* have a long history as singing pets in China. The complete mitochondrial genome of *Gampsocleis fletcheri* (Burr, 1899) is 15,719 bp in size. It consists of 13 protein-coding genes (PCGs), 22 tRNA genes, two rRNA genes, and an A + T-rich region. The base composition of *G. fletcheri* mitochondrial genome is A (34.7%), T (30.4%), G (12.0%), and C (22.9%), with an A + T bias (65.1%). Phylogenetic analyses based on the 13 PCGs showed that *G. fletcheri* was sister to *G. gratiosa*. The present genomic-level and male genitalia evidences support to restore the taxonomic status of *G. fletcheri*.

Species of *Gampsocleis* have a long history as singing pets in China. *Gampsocleis fletcheri* (Burr, 1899) was described as *Drymadusa fletcheri*. Chang (1935) transferred *D. fletcheri* to genus *Gampsocleis* and considered it as synonym of *Gampsocleis gratiosa gratiosa*. Through morphological comparison, we found that the male genitalia of *G. fletcheri* is significantly different from *G. gratiosa gratiosa* (Figure 1). Here, we elucidate the complete mitochondrial genome of *G. fletcheri*.

**Figure 1.**
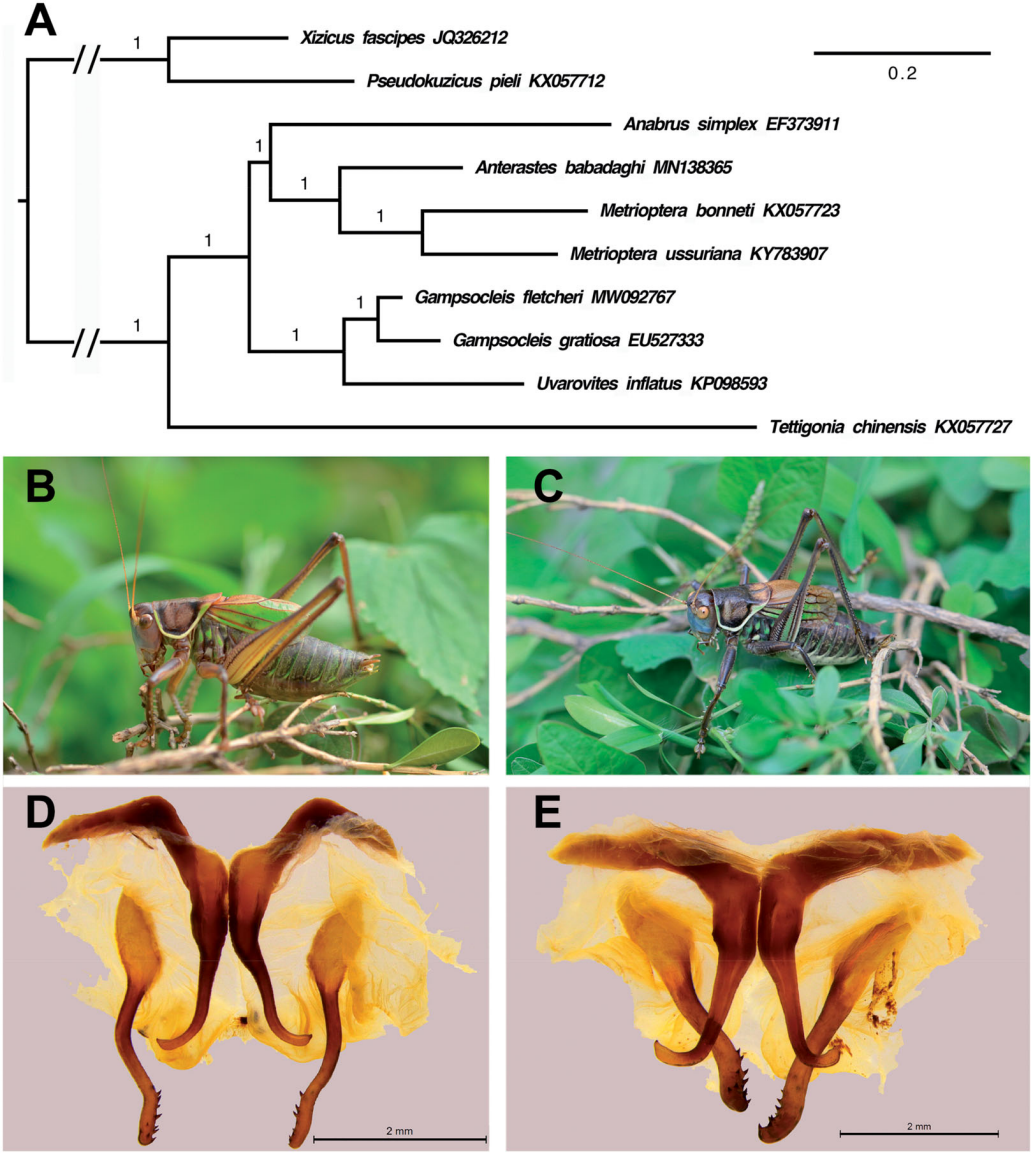
(A) Phylogenetic analysis among Tettigoniinae based on mitochondrial 13 PCGs. (B) Appearance of the adult male *G. fletcheri*. (C) Appearance of the adult male *G. gratiosa gratiosa.* (D) The male genitalia of *G. fletcheri*. (E) The male genitalia of *G. gratiosa gratiosa*.

The specimens of *G. fletcheri* were collected from Yantai (37.81°N, 120.76°E), Shandong province, China on August 2018 and immediately preserved in anhydrous ethanol. The voucher specimen was stored in the Museum of Hebei University with an accession number ZYX592. Total genomic DNA was extracted with 25 mg hindleg muscles of a single adult using the TIANamp Genomic DNA Kit (Tiangen Biotech, Beijing, China) and then sequenced using Illumina HiSeq 2500 platform. The mitochondrial genome was assembled and annotated using MitoZ (Meng et al. 2019).

The complete mitochondrial genome of *G. fletcheri* was a circular molecule of 15,719 bp in size (GenBank accession number: MW092767). It consists of 13 protein-coding genes (PCGs), 22 tRNAs, two rRNAs, and an A + T rich region. Four PCGs (*nad1*, *nad4*, *nad5*, and *nad4l*), eight tRNA genes (*trnQ*, *trnC*, *trnY*, *trnF*, *trnH*, *trnP*, *trnL(CUN)*, *trnV*), and two rRNA genes (*lrRNA* and *srRNA*) encoded on the N-strand, the remaining genes encoded on the J-strand. The composition of each base was as follows: A (34.71%), T (30.42%), G (11.94%), and C (22.93%), with a strong bias toward A + T (65.13%). All 13 PGCs initiated with ATN codon. Eleven out of 13 PCGs terminated with the stop codon TAA or TAG, whereas *atp6* terminated with incomplete terminal TA, *nad4* terminated with incomplete terminal T. The 22 tRNA genes range from 63 bp to 70 bp in length, all tRNA genes except for *trnS(AGN)* formed typical clover-leaf structure. The two rRNA genes (*lrRNA* and *srRNA*) were located between *trnL(CUN)* and *trnV*, and between *trnV* and the A + T-rich region, respectively. The lengths and A + T content of *lrRNA* and *srRNA* were determined to be 1363 bp and 777 bp, and 70.33% and 68.47%, respectively. The A + T-rich region was located in the conserved position between *srRNA* and *trnI*. The length and the A + T content of this region was 966 bp and 67.91%, respectively.

To determine the phylogenetic relationship of *G. fletcheri* with other seven Tettigoniinae species, we reconstructed the phylogenetic tree using MrBayes 3.1.2 (Ronquist et al. 2012) based on mitochondrial 13 PCGs sequences. Both *Xizicus fascipes* (JQ326212) and *Pseudokuzicus pieli* (KX057712) from Meconematinae were used as outgroups. The 13 PCGs sequences were aligned using MUSCLE algorithm in MEGA X (Kumar et al. 2018). Ambiguously aligned sites were excluded in Gblocks v.0.91b (Castresana 2000) and then concatenated using SequenceMatrix v1.7.8 (Vaidya et al. 2011). The optimal partitioning scheme for the concatenated data was searched using PartitionFinder v.2.1.1 (Lanfear et al. 2017). The result showed that *G. fletcheri* was sister to *G. gratiosa*, and the two species were further grouped with *U. inflatus*. Combining male genitalia and mitochondrial genome, we believed that the taxonomic status of *G. fletcheri* should be restored.

## Data Availability

The genome sequence data that support the findings of this study are openly available in GenBank of NCBI at https://www.ncbi.nlm.nih.gov/ under the accession no. MW092767. The associated ‘BioProject’, ‘SRA’, and ‘Bio-Sample’ numbers are PRJNA679154, SRR13083228, and SAMN16824904, respectively.
